# A Partial Least Squares based algorithm for parsimonious variable selection

**DOI:** 10.1186/1748-7188-6-27

**Published:** 2011-12-05

**Authors:** Tahir Mehmood, Harald Martens, Solve Sæbø, Jonas Warringer, Lars Snipen

**Affiliations:** 1 Biostatistics, Department of Chemistry, Biotechnology and Food Sciences, Norwegian University of Life Sciences, Norway; 2 Centre of Integrative Genetics (CIGENE), Animal and Aquacultural Sciences, Norwegian University of Life Sciences, Norway; 3 Department of Cell- and Molecular Biology, University of Gothenburg, Sweden

## Abstract

**Background:**

In genomics, a commonly encountered problem is to extract a subset of variables out of a large set of explanatory variables associated with one or several quantitative or qualitative response variables. An example is to identify associations between codon-usage and phylogeny based definitions of taxonomic groups at different taxonomic levels. Maximum understandability with the smallest number of selected variables, consistency of the selected variables, as well as variation of model performance on test data, are issues to be addressed for such problems.

**Results:**

We present an algorithm balancing the parsimony and the predictive performance of a model. The algorithm is based on variable selection using reduced-rank Partial Least Squares with a regularized elimination. Allowing a marginal decrease in model performance results in a substantial decrease in the number of selected variables. This significantly improves the understandability of the model. Within the approach we have tested and compared three different criteria commonly used in the Partial Least Square modeling paradigm for variable selection; loading weights, regression coefficients and variable importance on projections. The algorithm is applied to a problem of identifying codon variations discriminating different bacterial taxa, which is of particular interest in classifying metagenomics samples. The results are compared with a classical forward selection algorithm, the much used Lasso algorithm as well as Soft-threshold Partial Least Squares variable selection.

**Conclusions:**

A regularized elimination algorithm based on Partial Least Squares produces results that increase understandability and consistency and reduces the classification error on test data compared to standard approaches.

## Background

With the tremendous increase in data collection techniques in modern biology, it has become possible to sample observations on a huge number of genetic, phenotypic and ecological variables simultaneously. It is now much easier to generate immense sets of raw data than to establish relations and provide their biological interpretation [1-3]. Considering cases of supervised statistical learning, huge sets of measured/collected variables are typically used as explanatory variables, all with a potential impact on some response variable, e.g. a phenotype or class label. In many situations we have to deal with data sets having a large number of variables *p *in comparison to the number of samples *n*. In such 'large *p *small *n*' situations selection of a smaller number of influencing variables is important for increasing the performance of models, to diminish the curse of dimensionality, to speed up the learning process and for interpretation purposes [4,5]. Thus, some kind of variable selection procedure is frequently needed to eliminate unrelated features (noise) for providing a more observant analysis of the relationship between a modest number of explanatory variables and the response. Examples include the selection of gene expression markers for diagnostic purposes, selecting SNP markers for explaining phenotype differences, or as in the example presented here, selecting codon preferences discriminating between different bacterial phyla. The latter is particularly relevant to the classification of samples in metagenomic studies [6]. Multivariate approaches like correspondence analysis and principal component analysis has previously been used to analyze variations in codon usage among genes [7]. However, in order to relate the selection specifically to a response vector, like the phylum assignment, we need a selection based on a supervised learning method.

Partial Least Square (PLS) regression is a supervised method specifically established to address the problem of making good predictions in the 'large *p *small *n*' situation, see [8]. PLS in its original form has no implementation of variable selection, since the focus of the method is to find the relevant linear subspace of the explanatory variables, not the variables themselves. However, a very large *p *and small *n *can spoil the PLS regression results, as demonstrated by Keles *et. al. *[9], discovering that the asymptotic consistency of the PLS estimators for univariate responses do not hold, and by [10], who observed a large variation on test set.

Boulesteix has theoretically explored a tight connection between PLS dimension reduction and variable selection [11] and work in this field has existed for many years. Examples are [8,9,11-23]. For an optimum extraction of a set of variables, we need to look for all possible subsets of variables, which is impossible if *p *is large enough. Normally a set of variables with a reasonable performance is a compromise over the optimal sub set.

In general, variable selection procedures can be categorized [5] into two main groups: filter methods and wrapper methods. Filter methods select variables as a preprocessing step independently of some classifier or prediction model, while wrapper methods are based on some supervised learning approach [12]. Hence, any PLS-based variable selection is a wrapper method. Wrapper methods need some sort of criterion that relies solely on the characteristics of the data as described by [5,12]. One candidate among these criteria is the PLS loading weights, where down-weighting small PLS loading weights is used for variable selection [8,11,13-17,24-27]. A second possibility is to use the magnitude of the PLS regression coefficients for variable selection [18-20,28-34]. Jackknifing and/or bootstrapping on regression coefficients has been utilized to select influencing variables [20,30,31,33,34]. A third commonly used criterion is the Variables Importance on PLS projections (VIP) introduced by Eriksson *et. al. *[21] and is commonly used in practise [22,31,35-37].

There are several PLS-based wrapper selection algorithms, for example uninformative variable elimination (UVE-PLS) [18], where artificial random variables are added to the data as a reference such that the variable with least performance are eliminated. Iterative PLS (IPLS) adds new variable(s) in the model or remove variables from the model if it improves the model performance [19]. A backward elimination procedure based on leave one variable out is another example [5]. Although wrapper based methods perform well the number of variables selected is still often large [5,12,38], which may make interpretation hard ([23,39,40]).

Among recent advancements in PLS methodology itself we find that Indahl *et. al. *[41] propose a new data compression method for estimating optimal latent variables classification and regression problems by combining PLS methodology and canonical correlation analysis (CCA), called Canonical Powered PLS (CPPLS). In our work we have adopted this new methodology and proposed a regularized greedy algorithm based on a backward elimination procedure. The focus is on classification problems, but the same concept can be used for prediction problems as well. Our principle idea is to focus on a parsimonious selection, achieved by tolerating a minor performance deviation from any 'optimum' if this gives a substantial decrease in the number of selected variables. This is implemented as a regularization of the search for optimum performance, making the selection less dependent on 'peak performance' and hence more stable. In this respect, the choice of the CPPLS variant is not important, and even the use of non-PLS based methods could in principle be implemented with some minor adjustments. Both loading weights, PLS regression coefficients significance obtained from jackknifing and VIP are explored here for ordering the variables with respect to their importance.

## 1 Methods

### 1.1 Model fitting

We consider a classification problem where every object belongs to one out of two possible classes, as indicated by the *n *× 1 class label vector ***C***. From ***C ***we create the *n *× 1 numeric response vector ***y ***by dummy coding, i.e. ***y ***contains only 0's and 1's. The association between ***y ***and the *n *× *p *predictor matrix ***X ***is assumed to be explained by the linear model E(***y***) = ***X****β *where *β *are the *p *× 1 vector of regression coefficients. The purpose of variable selection is to find a column subset of ***X ***capable of satisfactory explaining the variations in ***C***.

From a modeling perspective, ordinary least square fitting is no option when *n *<*p*. PLS resolves this by searching for a small set of components, 'latent vectors', that performs a simultaneous decomposition of ***X ***and ***y ***with the constraint that these components explain as much as possible of the covariance between ***X ***and ***y***.

### 1.2 Canonical Powered PLS (CPPLS) Regression

PLS is an iterative procedure where relation between ***X ***and ***y ***is found through the latent variables. The PLS estimate of the regression coefficients for the above given model based on *k *components can be achieved by

(1) $$\hat{\beta }=\hat{W}{\text{(}{\hat{P}}_{1}^{'}\hat{W})}^{-1}{\hat{p}}_{2}^{'}$$

where ${\hat{P}}_{1}$ is the *p*_l _× *k *matrix of ***X***-loadings that is summary of X-variables, ${\hat{p}}_{2}$ is the *a *vector of ***y***-loadings i.e. summary of y-variables and $\hat{W}$ is the *p *× *k *matrix of loading weights, for details see [8]. Recently, Indahl *et. al. *[41] proposed a new data compression method for estimating optimal latent variables by combining PLS methodology and canonical correlation analysis (CCA). They introduce a flexible trade-off between the element wise correlations and variances specified by a power parameter *γ*, ranging from 0 to 1. Defines the loading weights as

$$\begin{array}{c} w\left(\gamma \right)=K_{\gamma }\left[s_{1}|corr\left(x_{1},y\right){|}^{\frac{\gamma }{1-\gamma }}\right).std{\left(x_{1}\right)}^{\frac{1-\gamma }{\gamma }},...,\\ \;\;\;\;\;\;\;\;\;\;\;\;\;\;\;\;\;\;\;\;\;\;\;\;{\left(s_{p}|corr\left(x_{p},y\right){|}^{\frac{\gamma }{1-\gamma }}.std{\left(x_{p}\right)}^{\frac{1-\gamma }{\gamma }}\right]}^{t} \end{array}$$

where *s*_*k *_denotes the sign of the *k*^*th *^correlation and *K*_*γ *_is a scaling constant assuring unit length *w*(*γ*). In this study we restricted *γ *to lower region (0.001, 0.050) and to upper region (0.950, 0.999). This means we consider combinations of *γ *for emphasizing either the variance (*γ *close to 0) or the correlations (*γ *close to 1). The *γ *value from above regions that optimizes the canonical correlation is always selected for each component of CPPLS algorithm, see Indahl *et. al. *[41] for details on the CPPLS algorithm.

Based on the CPPLS estimated regression coefficients $\hat{\beta }$ we can predict the dummy-variables by

$$\hat{y}=X\hat{\beta }$$

and from the data set $\left(\hat{y},C\right)$ we build a classifier using straightforward linear discriminant analysis [42].

### 1.3 First regularization - model dimension estimate

The CPPLS algorithm assumes that the column space of ***X ***has a subspace of dimension *α *containing all information relevant for predicting ***y ***(known as the relevant subspace) [43]. In order to estimate *α *we use cross-validation and the performance *P*_*a *_defined as the fraction of correctly classified observations in a cross-validation procedure, using *a *components in the CPPLS algorithm.

The cross-validation estimate of *α *can be found by systematically trying out a range of dimensions *a *= 1,..., *A*, compute *P*_*a *_for each *a*, and choose as $\hat{\alpha }$ the *a *where we reach the maximum *P*_*a*_. Let us denote this value a*. It is well known that in many cases *P*_*a *_will be almost equally large for many choices of *a*. Thus, estimating *α *by this maximum value is likely to be a rather unstable estimator. To stabilize this estimate we use a regularization based on the principle of parsimony where we search for the smallest possible *a *whose corresponding performance is not significantly worse than the optimum. If *ρ*_*a *_is the probability of a correct classification using the *a*-component model, and *ρ*_a* _similar for the a*-component model, we test *H*_0 _: *ρ*_*a *_= *ρ*_a* _against the alternative *H*_1 _: *ρ*_*a *_<*ρ*_a*_. In practice *P*_*a *_and *P*_a* _are estimates of *ρ*_*a *_and *ρ*_a*_. The smallest *a *where we cannot reject *H*_0 _is our estimate $\hat{\alpha }$. The testing is done by analyzing the 2 × 2 contingency table of correct and incorrect classifications for the two choices of *a*, using the McNemar test [44]. This test is appropriate since the model classification at a specific component depends on the model classification at the other components.

This regularization depends on a user-defined rejection level *c *of the McNemar test. Using a large *c *(close to 1) means we easily reject *H*_0_, and the estimate $\hat{\alpha }$ is often similar to a*. By choosing a smaller *c *(closer to 0) we get a harder regularization, i.e. a smaller $\hat{\alpha }$ and more stability at the cost of a lower performance.

### 1.4 Selection criteria

We have implemented and tried out three different criteria for PLS-based variable selection:

#### 1.4.1 Loading weights

Variable *j *can be eliminated if the relative loading weight, *r*_*j *_for a given PLS component satisfies $r_{j}=|\frac{w_{a,j}}{\max w_{a}}|<u$ for some chosen threshold *u *∈ [0, 1].

#### 1.4.2 Regression coefficients

Variable *j *can be eliminated if the corresponding regression coefficient *β*_*j *_= 0. Testing *H*_0 _: *β*_*j *_= 0 against *H*_1_: *β*_*j *_≠ = 0 can be done by a jackknife t-test. All computations needed have already been done in the cross-validation used for estimating the model dimension *α*. For each variable we compute the corresponding false discovery rate (*q *-value) which is based on the *p *values from jackknifing, and variable *j *can be eliminated if *q*_*j *_>*u *for some fixed threshold *u *∈ [0, 1].

#### 1.4.3 Variable importance on PLS projections (VIP)

VIP for the variable *j *is defined according to [21] as

$$v_{j}=\sqrt{p\sum_{a=1}^{a*}[(p_{2a}^{2}{t^{\prime }}_{a}t_{a}){\left(w_{aj}/||w_{a}||\right)}^{2}]/\sum_{a=1}^{a*}(p_{2a}^{2}({t^{\prime }}_{a}t_{a})}$$

where *a *= 1, 2, ..., a*, *w*_*aj *_is the loading weight for variable *j *using *a *components and ***t***_*a*_, ***w***_*a *_and *p*_2*a *_are CPPLS scores, loading weights and *y*-loadings respectively corresponding to the *a*^*th *^component. [22] explains the main difference between the regression coefficient *β*_*j *_and *v*_*j*_. The *v*_*j *_weights the contribution of each variable according to the variance explained by each PLS component, i.e. $p_{2a}^{2}t_{a}^{\prime }t_{a}$ where (***w***_*aj*_/||***w***_*a*_||)^2 ^represents the importance of the *j*^*th *^variable. Variable *j *can be eliminated if *v*_*j *_<*u *for some user-defined threshold *u *∈ [0, ∞). It is generally accepted that a variable should be selected if *v*_*j *_> 1, see [21,22,36], but a proper threshold between 0.83 and 1.21 can maximize the performance [36].

### 1.5 Backward elimination

When we have *n *<<*p *it is very difficult to find the truly influencing variables since the estimated relevant subspace found by Cross-Validated CPPLS (CVCPPLS) is bound to be, to some degree, 'infested' by non-influencing variables. This may easily lead to errors both ways, i.e. both false positives and false negatives. An approach to improve on this is to implement a stepwise estimation where we gradually eliminate 'the worst' variables in a greedy algorithm.

The algorithm can be sketched as follows: Let ***Z***_0 _= ***X ***and let *s*_*j *_be one of the criteria for variable *j *we have sketched above (either *r*_*j*_, *q*_*j *_or *v*_*j*_).

1) For iteration *g *run ***y ***and ***Z***_*g *_through CVCPPLS. The matrix ***Z***_*g *_has *p*_*g *_columns, and we get the same number of criterion values, sorted in ascending order as $s_{\left(1\right)},...,s_{\left(p_{g}\right)}$.

2) There are *M *criterion values below (above for citerion *q*_*j*_) the cutoff *u*. If *M *= 0, terminate the algorithm here.

3) Else, let *N *= ⌈*fM*⌉ for some fraction *f *∈ 〈0,1]. Eliminate the variables corresponding to the *N *most extreme criterion values.

4) If there are still more than one variable left, let ***Z***_*g*+1 _contain these variables, and return to 1).

The fraction *f *determines the 'steplength' of the elimination algorithm, where an *f *close to 0 will only eliminate a few variables in every iteration. The fraction *f *and *u *can be obtained through cross validation.

### 1.6 Second regularization - final selection

In each iteration of the elimination the CVCPPLS algorithm computes the cross-validated performance, and we denote this with *P*_*g *_for iteration *g*. After each iteration, the number of influencing variables decreases, but *P*_*g *_will often increase until some optimum is achieved, and then drop again as we keep on eliminating. The initial elimination of variables stabilizes the estimates of the relevant subspace in the CVCPPLS algorithm, and hence we get an increase in performance. Then, if the elimination is too severe, we start to lose informative variables, and even if stability is increased even more, the performance drops.

Let the optimal performance be defined as

$$P^{*}=P_{g^{*}}=\underset{g}{\max}P_{g}$$

**Figure 1:**
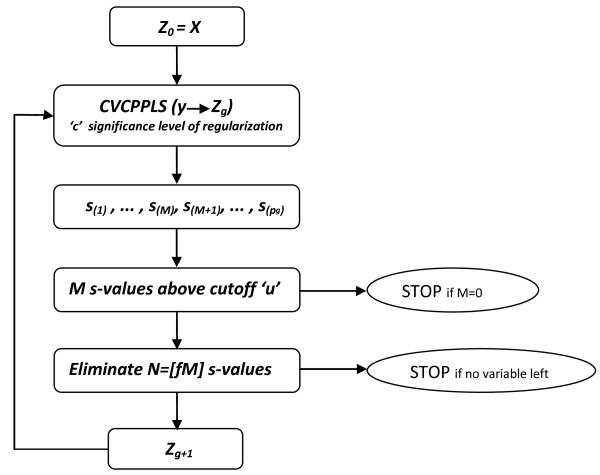
**Flow chart**. The flow chart illustrates the proposed algorithm for variable selection.

It is not unreasonable to use the variables still present after iteration g* as the final selected variables. This is where we have achieved a balance between removing noise and keeping informative variables. However, frequently we observe that a severe reduction in the number variables compared to this 'optimum' will give only a modest drop in performance. Hence, we may eliminate well beyond g*, and find a much simpler model, at a small loss in performance. To formalize this, we use exactly the same procedure, involving the McNemar test that we used in the regularization of the model dimension estimate. If *ρ*_*g *_is the probability of a correct classification after *g *iterations, and *ρ*_g* _similar after g* iterations, we test *H*_0 _: *ρ*_*g *_= *ρ*_g* _against the alternative *H*_1 _: *ρ*_*g *_<*ρ*_g*_. The largest *g *where we cannot reject *H*_0 _is the iteration where we find our final selected variables. This means we need another rejection level *d *which will decide to which degree we are willing to sacrifice performance over a simpler model. Using *d *close to 0 means we emphasize simplicity over performance. In practice, for each iteration beyond g* we can compute the McNemar test *p-*value, and list this together with the number of variables remaining, to give a perspective on the trade-off between understandability of the model and the performance. Figure 1 presents the procedure in a flow chart.

### 1.7 Choices of variable selection methods for comparison

Three variable selection methods are also considered for comparison purposes. The classical forward selection procedure (Forward) is a univariate approach, and probably the simplest approach to variable selection for the 'large p small n' type of problems considered here. The Least Absolute Shrinkage and Selection Operator (Lasso) [45] is a method frequently used in genomics. Recent examples are the extraction of molecular signatures [46] and gene selection from microarrays [47]. The Soft-Thresholding PLS (ST-PLS) [17] implements the Lasso concept in a PLS framework. A recent application of ST-PLS is the mapping of genotype to phenotype information [48].

All methods are implemented in the R computing environment http://www.r-project.org/.

## 2 Application

An application of the variable selection procedure is to find the preferred codons associated with certain prokaryotic phyla.

Codons are triplets of nucleotides in coding genes and the messenger RNA; these triplets are recognized by base-pairing by corresponding anticodons on specific transfer RNA carrying individual amino acids. This facilitates the translation of genetic messenger information into specific proteins. In the standard genetic code, the 20 amino acids are individually coded by 1, 2, 4 or 6 different codons (excluding the three stop codons there are 61 codons). However, the different codons encoding individual amino acids are not selectively equivalent because the corresponding tRNAs differ in abundance, allowing for selection on codon usage. Codon preference is considered as an indicator of the force shaping genome evolution in prokaryotes [49,50], reflection of life style [49] and organisms within similar ecological environments often have similar codon usage pattern in their genomes [50,51]. Higher order codon frequencies, e.g. di-codons, are considered important with respect to joint effects, like synergistic effect, of codons [52].

There are many suggested procedures to analyze codon usage bias, for example the codon adaptation index [53], the frequency of optimal codons [54] and the effective number of codons [55]. In the current study, we are not specifically looking at codon bias, but how the overall usage of codons can be used to distinguish prokaryote phyla. Notice that the overall codon usage is affected both by the selection of amino acids and codon bias within the redundant amino acids. Phylum is a relevant taxonomic level for metagenomic studies [56,57], so interest lies in having a systematic search for codon usage at the phylum level [58-60].

### 2.1 Data

Genome sequences for 445 prokaryote genomes and the respective Phylum information were obtained from NCBI Genome Projects (http://www.ncbi.nlm.nih.gov/genomes/lproks.cgi). The response variable in our data set is phylum, i.e. the highest level taxonomic classifier of each genome, in the bacterial kingdom. There are in total 11 several phyla in our data set including *Actinobacteria*, *Bacteroides*, *Crenarchaeota*, *Cyanobac-teria*, *Euryarchaeota*, *Firmicutes*, *Alphaproteobacteria*, *Betaproteobacteria*, *Deltaproteobacteria*, *Gammapro-teobacteria *and *Epsilonproteobacteria*. We only consider two-class problems, i.e. for some fixed 'phylum A', we only classify genomes as either 'phylum A', or 'not phylum A'. Thus, the data set has *n *= 445 samples and 11 different responses of 0/1 outcome, considering one at a time.

Genes for each genome were predicted by the gene-finding software Prodigal [61], which uses dynamic programming in which start codon usage, ribosomal site motif usage and GC frame bias are considered for gene prediction. For each genome, we collected the frequencies of each codon and each di-codon over all genes. The predictor variables thus consists of relative frequencies for all codons and di-codons, giving a predictor matrix ***X ***with a total of *p *= 64 + 64^2 ^= 4160 variables (columns).

### 2.2 Parameter setting/tuning

It is in principle no problem to eliminate (almost) all variables, since we always go back to the iteration where we cannot reject the null-hypothesis of the McNemar test. Hence, we fixed *u *at extreme values, 0.99 for loading weights, 0.01 for regression coefficients and 10 for VIP. Three levels of step length *f *= (0.1,0.5, 1) were considered. In the first regularization step we tried three very different rejection levels *c *= (0.1,0.5, 1) and in the second we used two extreme levels (*d *= (0.01,0.99)).

### 2.3 The split of data into test and training

**Figure 2:**
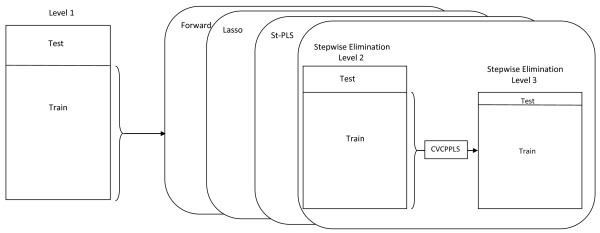
**An overview of the testing/training**. An overview of the testing/training procedure used in this study. The rectangles illustrate the predictor matrix. At level 1 we split the data into a test set and training set (25/75) to be used by all four methods listed on the right. This was repeated 100 times. Inside our suggested method, the stepwise elimination, there are two levels of cross-validation. First a 10-fold cross-validation was used to optimize selection parameters *f *and *d*, and at level 3 leave-one-out cross-validation was used to optimize the regularized CPPLS method.

Figure 2 gives a graphical overview of the data splitting used in this study. The split is carried out at three levels. At level 1 we split the data into a test set containing 25% of the genomes and a training set containing the remaining 75%. This was repeated 100 times, i.e. 100 pairs of test and training sets were constructed by random drawing with replacement. Test and training set were never allowed to overlap. In each of the 100 instances, the training data were used by each of the four methods listed to the right. They select variables, and the selected variables were used for classifying the level 1 test set, and performance was computed for each method.

Inside our suggested method, the stepwise elimination, there are two levels of cross-validation as indicated by the right part of the figure. First, a 10-fold cross-validation was used to optimize the fraction *f *and the rejection level *d *in the elimination part of our algorithm. At level 3 leave-one-out cross-validation was used to estimate all parameters in the regularized CPPLS method, including the rejection level *c*. These two levels together corresponds to a 'cross-model validation' [62].

## 3 Results and Discussions

**Figure 3:**
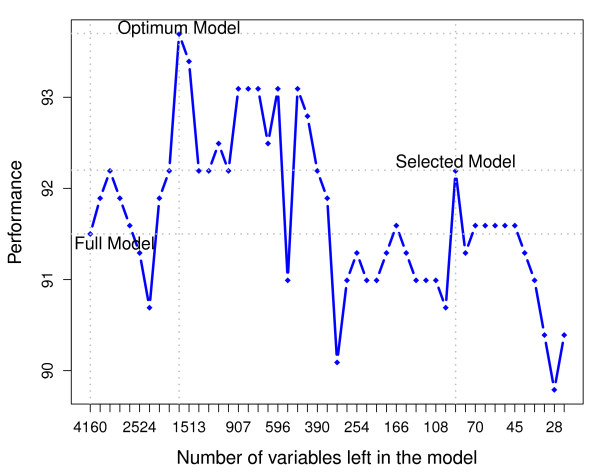
**A typical elimination**. A typical elimination is shown based on the data for phylum *Actinobacteria*. Each dot in the figure indicates one iteration. The procedure starts on the left hand side, with the full model. After some iterations performance(P), which reflects the percentage of correctly classified samples, has increased, and reaches a maximum. Further elimination reduces performance, but only marginally. When elimination becomes too severe, the performance drops substantially. Finally, the selected model is found where we have the smallest model with performance not significantly worse than the maximum.

For identification of codon variations that distinguishes different bacterial taxa to be utilized as classifiers in metagenomic analysis, 11 models, representing each phylum, were considered separately. We have chosen the phylum *Actinobacteria *for a detailed illustration of the method, while results for all phyla are provided below. In Figure 3 we illustrate how the elimination process affects model performance (P) reflecting the percentage of correctly classified samples, starting at the left with the full model and iterating to the right by eliminating variables. Use of any of the three criteria loading weights, regression coefficient significance or VIP, produces the same type of behavior. A fluctuation in performance over iterations is typical, reflecting the noise in the data. At each iteration, we only eliminate a fraction (1% in many cases) of the poor ranking variables, giving the remaining variables a chance to increase the performance at later iterations. We do not stop at the maximum performance, which may occur almost anywhere due to the noise, but keep on eliminating until we reach the minimum model not significantly worse than the best. This may lead to a substantial decrease in the number of variables selected.

**Figure 4:**
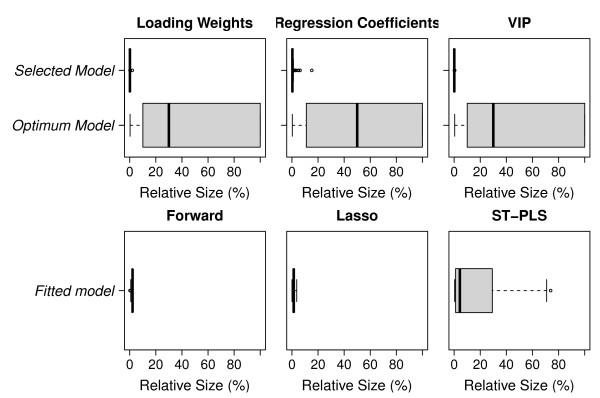
**The distribution of selected variables**. The distribution of the number of variables selected by the optimum model and selected model for loading weights, VIP and regression coefficients is presented in upper panels, while lower panels display similar for Forward, Lasso and ST-PLS. The horizontal axes are the number of retained variables as percentage of the full model (with 4160 variables). All results are based on 100 random samples from the full data set, where 75% of the objects are used as training data and 25% as test data in each sample.

In the upper panels of Figure 4, a comparison of the number of variables selected by the 'optimum' model and our selected model is displayed. A cross-comparison of the criteria loading weights, regression coefficient and VIP based elimination procedure is also made. Xiaobo *et. al. *[23] has criticized the wrapper procedures for being unable to extract a small number of variables, which is important for interpretation purposes [23,39]. This is reflected here as none of the 'optimum' model selections (lower boxes) resulted in a small number of selected variables. However, using our regularized algorithm (upper boxes) we are able to select a small number of variables in all cases. The VIP based elimination performs best in this respect (upper right panel), but the other criteria are also competing well. The variance in model size is also very small for our regularized algorithm compared to the selection based on 'optimum' performance.

Comparison of the number of variables selected by Forward, Lasso and ST-PLS is made in the lower panels of Figure 4. All three methods end up with a small number of selected variables on the average, but ST-PLS has a large variation in the number of selected variables.

**Figure 5:**
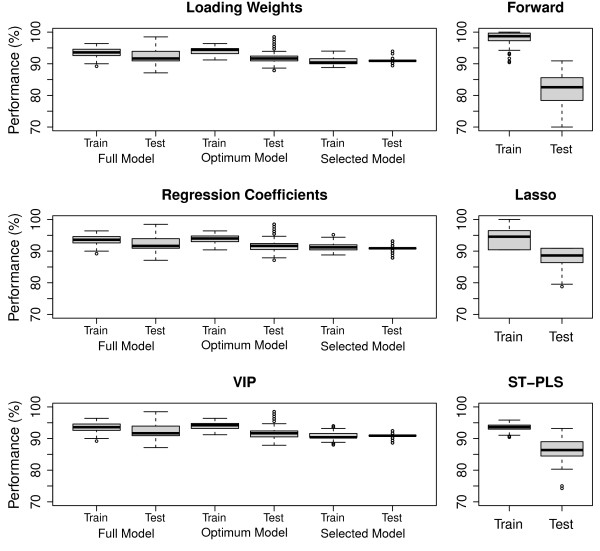
**Performance comparison**. The left panel presents the distribution of performance of in the full model, optimum model and selected models on test and training data sets for loading weights, VIP and regression coefficients, while the right panels display similar for Forward, Lasso and ST-PLS. All results are based on 100 random samples from the full data set, where 75% of the objects are used as training data and 25% as test data in each sample.

The classification performances in the test and training data sets are examined in Figure 5. In the left panels we show the results for our procedure using the criteria loading weights, regression coefficient and VIP during selection. In general all three criteria behave equally well. As expected, the best performance is found in the training data for the 'optimum' model. Also, performance is consistently worse in the test data compared to the training data for all cases, but this pattern is most clearly present for the 'optimum' model. This may be seen as an indication of over-fitting. A huge variation in test data performance can be observed for the full model, and slightly smaller for the 'optimum' model. Our selected models give somewhat worse overall training performance, but evaluated on the test sets they come out at the same level as the 'optimum' model, and with a much smaller variance.

In the right hand panels, performance is shown for the three alternative methods. Our algorithm comes out with at least as good performance on the test sets as any of the three alternative methods. Particularly notably is the larger variation in test performance for the alternative methods compared to the selected models in the left panels. A formal testing by the Mann-Whitney test [63] indicates that our suggested procedure with VIP outperforms Lasso (*p *< 0.001), Forward (*p *< 0.001) and ST-PLS (*p *< 0.001) on the data sets used in this study. The same was also true if we used loading weights or regression coefficient as ranking criteria.

**Figure 6:**
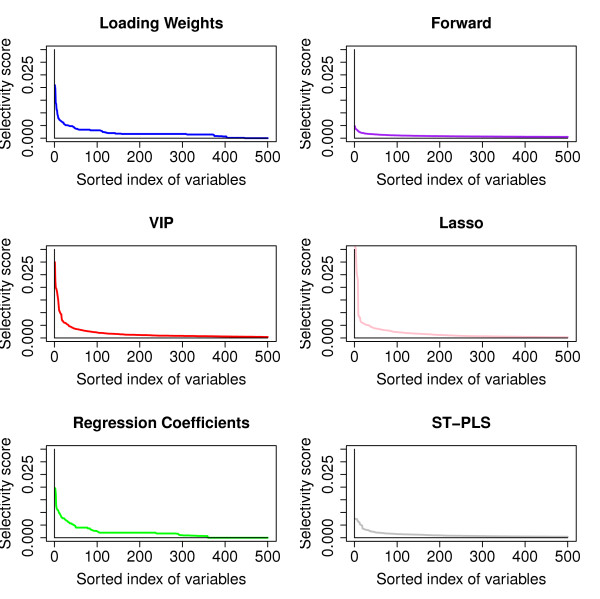
**Selectivity score**. The selectivity score is sorted in descending order for each criterion loading weights, regression coefficients significance and VIP in the left panels, while right panels display similar for Forward, Lasso and ST-PLS. Only the first 500 values (out of 4160) are shown.

When we are interested in the interpretation of the variables, it is imperative that the procedure we use shows some stability with respect to which variables are being selected. To examine this we introduce a *selectivity score*. If a variable is selected as one out of *m *variables, it will get a score of 1*/m*. Repeating the selection 100 times for the same variables, but with slightly different data, we add up the scores for each variable and divide by 100. Thus, a variable having a large selectivity score is often selected as one among a few variables. A procedure that selects almost all variables, or completely new variables, each time, will produce very similar and small selectivity scores for all variables. Conversely, a procedure that home in on the same few variables in each case, will produce some very big selectivity scores on these variables. In Figure 6 we show the selectivity scores sorted in descending order for the three criteria loading weights, regression coefficients and VIP, and for the alternative methods Forward, Lasso and ST-PLS selection. This indicates that VIP is the most stable criterion, giving the largest selectivity scores, but loading weights and regression coefficient performs almost as good. The Lasso method is as stable as our proposed method using the VIP criterion, while Forward and ST-PLS seems worse as they spread the selectivity score over many more variables. From the definition of VIP we know that the importance of the variables is down-weighted as number of CPPLS components increases. This probably reduces the noise influence and thus provides more stable and consistent selection, also observed by [22].

95% of our selected model uses 1 component while the rest uses 2 components. It is clear from the definition of Loading weights, VIP and regression coefficients that the sorted index of variables based on these measures will be the same for 1 component. This could be the reason for the rather similar behavior of loading weights, VIP and regression coefficient in above analysis.

In order to get a rough idea of the 'null-distribution' of this selectivity score, we ran the selection on data where the response ***y ***was permuted at random. From this the upper 0.1% percentile of the null-distribution is determined, which is approximately corresponds to the selectivity score above 0.01. For each phylum and variables giving a selectivity score above this percentile are listed in Table 1. The selected variables will also have positive or negative impact depending on the sign of the regression coefficients as indicated in the table. A di-codon with a positive/negative regression coefficient is informative because it occurs more/less frequently in this phylum than in the entire population. It appears that, the larger phyla are in general more difficult to classify, simply because there are more diversity inside the group. On the other hand, the results obtained for the larger phyla are more relevant. Because a larger set of genomes usually means less sampling bias, i.e. the data set represents the phylum better. Interestingly, all of the selected variables are di-codons (no single codons), providing additional support for that the interaction of codons are highly important for explaining variations in phyla [49,52,64]. It should be noted that the performance listed for each phylum in Table 1 is an optimistic estimate of the real performance we must expect on a new data set, since it is based on variables selected by maximizing performance over all data in the present data set. However, for comparisons between phyla they are still relevant.

**Table 1:** Selectivity score based selected codons

Phylum	**Gen**.	**Perf**.	Positive and Negative impact
*Actinobacteria*	42	90.6	TCCGTA, TACGGA, GTGAAG, CTTCAC, TGTACA, TCCGTT, AGAAGG, CCTTCT, GAGGCT, GGAACA, TCCACC, TGTTCC, TTCCGT, CTTAAG, GGGATC, GATCCC, CCTTAA, TTAAGG AACGGA, GGTGGA, GTCGAC,
*Bacteroides*	16	96.3	TATATA, TCTATA, CTATAT, TATAGA, ATATAG, TATAGT, TTATAG, CTTATA, CTATAA, ACTATA, TATATC, GATATA, CTATAG, TATACT TATAAG, ATATAT,
*C renarchaeota*	16	96.5	AACGCT, AGCGTT, ACGAGT, ACTCGT, ACGACT, TTAGGG, TCGTGT, ACACGA, CCCTAA, TAGCGT, TACGAG, ACGCTA, CGTGTT, AACACG, GGGCTA, CTACGA, TCGTAG, CGAGTA, TACTCG, GCGTTT AGTCGT, CTCGTA, TAGCCC,
*Cyanobacteria*	17	97.1	CAATTG, GTTCAA, TTGAAC, TAAGAC, GTCTTA, CTTAGT, TTAGTC, GGTCAA, GACTAA, ACTAAG, CTTGAT, AAGTCA, ATCAAG, TGGTTC, GAACCA, AGTCAA, GACCAA, TTGGTC, TTGATC, GACTTG, TCTTAG, CAAGTC TTGACC, TGACTT, TTGACT, GATCAA,
*Euryarchaeota*	31	93.3	ACACCG, CGGTGT, TCGGGT, GGTGTC, TCGGTG, CACCGA, ACCCGA, CCGCGG, GGTGTG, TCACCG, TATCGT, TACGCT, TTCTGC GACACC, CACACC,
*Firmicutes*	89	80.3	TCGGTA, TACCGA, ACAGGA, TCCTGT
*Alphaproteobacteria*	70	85.9	TCGCGA, AAGATC, GATCTT, TTCGCG, AAATTT, CGCGAA
*Betaproteobacteria*	42	90.8	GGAACA, TGTTCC, TAGTCG, CGACTA, GCTAGC, AAGCTC, GAGCTT, TACGAG, CTCGTA, CTTGCA, GATCTT, TGCAAG, AAGATC, AGGCTT, AAGCCT, CTCGAG
*Gammaproteobacteria*	92	81.2	CTCAGT, ACTGAG, GACTCA, TGAGTC, ACTCAG, ACTCTG, CAGAGT, CTCAGA, TCTGAG, CTGTCT, CCAGAG, CTCTGG, TCACCT, TGACTC, CTCTGT, AGGTGA, GAGTCA, TCACTC, GAGTGA CTGAGT, AGACAG, ACAGAG,
*Deltaproteobacteria*	18	96.0	GACATT, TCATGT, ACATGA, AATGTC, AACATC, ATGTTG, CAACAT, CATTGT, ACAATG, ACATTG, ACAACA, TGTTGT, AACAAC, GTTGTT, CATTTC, GTTCCA, TGGAAC, CAACAA, TTGTTG, GAAACA, GGAACA, TGTTCC, AATGAC, GTCATT GATGTT, CAATGT, GAAATG, TGTTTC,
*Epsilonproteobacteria*	12	96.9	TCCTGT, ACAGGA, GTATCC, TCAGGA, TCCTGA, TGCAGA, TCTGCA, TTCAGG, CCTGAA, ATATCC, GAACCT, AGGTTC, GGAGAT, ATCTCC, TTGCAG, GGATAC, GGATAT, CTGCAA, TCCCTG, CAGGGA, ACTGCA, TGCAGT, TTCCTG, TACAGG

Results obtained for each phylum by using the VIP criterion. Gen. is the number of genomes for that phylum in the data set, Perf. is the average test-set performance i.e. percentage of correctly classified samples, when classifying the corresponding phylum. This is synonymous to the true positive rate. Positive impact variables are variables with selectivity score above 0.01 and with positive regression coefficients while Negative impact variables are similar with negative regression coefficients.

## 4 Conclusion

We have suggested a regularized backward elimination algorithm for variable selection using Partial Least Squares, where the focus is to obtain a hard, and at the same time stable, selection of variables. In our proposed procedure, we compared three PLS-based selection criteria, and all produced good results with respect to size of selected model, model performance and selection stability, with a slight overall improvement for the VIP criterion. We obtained a huge reduction in the number of selected variables compared to using the models with optimum performance based on training. The apparent loss in performance compared to the optimum based models, as judged by the fit to the training set, is virtually disappearing when evaluated on a separate test set. Our selected model performs at least as good as three alternative methods, Forward, Lasso and ST-PLS, on the present test data. This also indicates that the regularized algorithm not only obtain models with superior interpretation potential, but also an improved stability with respect to classification of new samples. A method like this could have many potential uses in genomics, but more comprehensive testing is needed to establish the full potential. This proof of principle study should be extended by multi-class classification problems as well as regression problems before a final conclusion can be drawn. From the data set used here we find a smallish number of di-codons associated with various bacterial phyla, which is motivated by the recognition of bacterial phyla in metagenomics studies. However, any type of genome-wide association study may potentially benefit from the use of a multivariate selection method like this.

## Competing interests

The authors declare that they have no competing interests.

## Authors' contributions

TM and LS initiated the project and the ideas. All authors have been involved in the later development of the approach and the final algorithm. TM has done the programming, with some assistance from SS and LS. TM and LS has drafted the manuscript, with inputs from all other authors. All authors have read and approved the final manuscript.
